# A metal-poor star with abundances from a pair-instability supernova

**DOI:** 10.1038/s41586-023-06028-1

**Published:** 2023-06-07

**Authors:** Qian-Fan Xing, Gang Zhao, Zheng-Wei Liu, Alexander Heger, Zhan-Wen Han, Wako Aoki, Yu-Qin Chen, Miho N. Ishigaki, Hai-Ning Li, Jing-Kun Zhao

**Affiliations:** 1 CAS Key Laboratory of Optical Astronomy, National Astronomical Observatories, Chinese Academy of Sciences, Beijing, China; 2 School of Astronomy and Space Science, University of Chinese Academy of Sciences, Beijing, China; 3 Yunnan Observatories, Chinese Academy of Sciences, Kunming, China; 4 Key Laboratory for the Structure and Evolution of Celestial Objects, Chinese Academy of Sciences, Kunming, China; 5 School of Physics and Astronomy, Monash University, Clayton, Victoria Australia; 6 Australian Research Council Centre of Excellence for All Sky Astrophysics in 3 Dimensions (ASTRO 3D), Sydney, New South Wales, Australia; 7 National Astronomical Observatory of Japan (NAOJ), Mitaka, Japan; 8 Astronomical Science Program, The Graduate University for Advanced Studies (SOKENDAI), Mitaka, Japan

**Keywords:** Stars, Stellar evolution

## Abstract

The most massive and shortest-lived stars dominate the chemical evolution of the pre-galactic era. On the basis of numerical simulations, it has long been speculated that the mass of such first-generation stars was up to several hundred solar masses^1–4^. The very massive first-generation stars with a mass range from 140 to 260 solar masses are predicted to enrich the early interstellar medium through pair-instability supernovae (PISNe)^5^. Decades of observational efforts, however, have not been able to uniquely identify the imprints of such very massive stars on the most metal-poor stars in the Milky Way^6,7^. Here we report the chemical composition of a very metal-poor (VMP) star with extremely low sodium and cobalt abundances. The sodium with respect to iron in this star is more than two orders of magnitude lower than that of the Sun. This star exhibits very large abundance variance between the odd- and even-charge-number elements, such as sodium/magnesium and cobalt/nickel. Such peculiar odd–even effect, along with deficiencies of sodium and α elements, are consistent with the prediction of primordial pair-instability supernova (PISN) from stars more massive than 140 solar masses. This provides a clear chemical signature indicating the existence of very massive stars in the early universe.

## Abstract (web summary)

The chemical composition of the Galactic halo star J1010+2358 shows extremely low sodium and cobalt abundances, different from most other halo stars, indicative of a very metal-poor star being seeded with elements from a pair-instability supernova.

## Main

The Galactic halo star LAMOST J1010+2358 (hereafter, J1010+2358, V-band magnitude *V* = 16.01) was identified as a VMP star to have a relatively low Mg abundance based on the Large Sky Area Multi-Object Fiber Spectroscopic Telescope (LAMOST) survey^8,9^. The analysis of the high-resolution spectrum from follow-up observation with the Subaru Telescope (Methods) confirms that J1010+2358 is a VMP star ([Fe/H] = −2.42) with extremely low α-element abundances (for example, [Mg/Fe] = −0.66). More than 400 VMP stars have been identified from the LAMOST survey and follow-up observations with high-resolution spectra^10,11^. None of these VMP stars exhibits such low α-element abundances. The remarkably low α elements to iron ratios, along with the unusual absence of sodium and barium, indicate that J1010+2358 may have recorded a chemical enrichment history completely different from those of most halo stars.

**Fig. 1:**
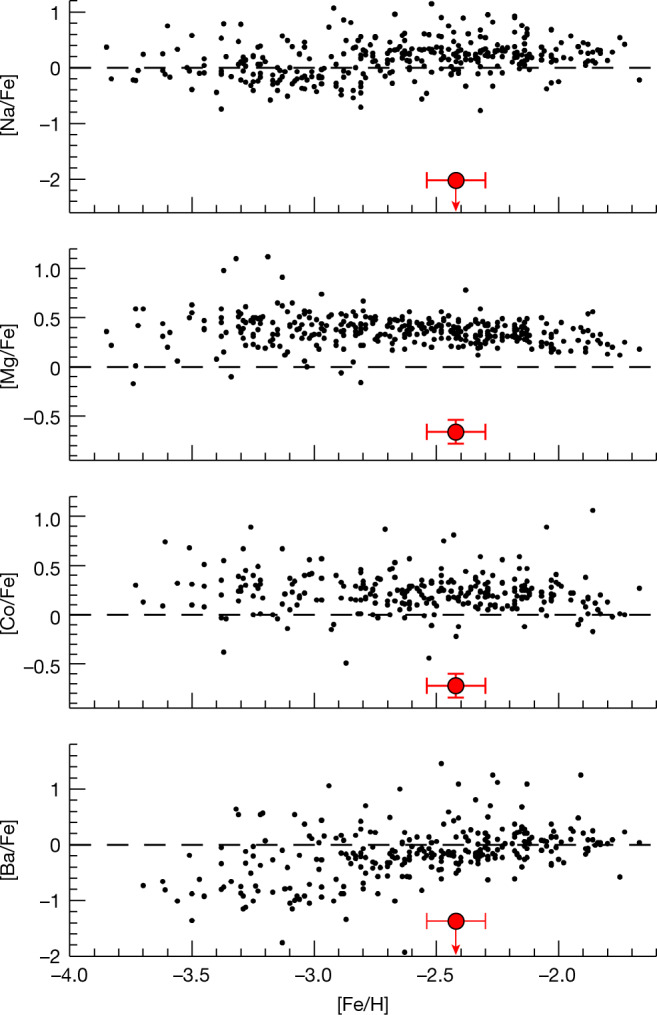
The abundances of J1010+2358 in comparison with those of other metal-poor stars. J1010+2358 is shown as the red circles. The black circles indicate the metal-poor stars from the literature^10,11^. The arrows indicate the upper limits. The error bars are 1*σ* uncertainties of the observed abundances.

The abundances of Mg, Si, Ca, Ti, Cr, Mn, Fe, Co and Ni shown in Table 1 are determined from the equivalent widths (EWs) based on one-dimensional plane-parallel local thermodynamic equilibrium (LTE) model atmospheres^12^. The upper limits of Na, Sc, Zn, Sr and Ba abundances are estimated by the spectrum synthesis method. As a VMP star with [Fe/H] = −2.42, the chemical abundances of J1010+2358 are very peculiar in comparison with other metal-poor stars in the Milky Way. This star has sub-solar [X/Fe] ratios for Na, Mg, Ca, Ti, Cr, Mn, Co, Ni and Zn. Its Na to Fe ratio ([Na/Fe] < −2.02) is lower than 1/100th of the solar value ^29^, whereas almost all other metal-poor stars exhibit Na/Fe ratios ([Na/Fe] > −1) greater than 1/10th of the solar value (Fig. 1). Furthermore, the Mg to Fe ratio of J1010+2358 ([Mg/Fe] = −0.66) is substantially lower than the typical abundance ratio of the Galactic halo stars with similar metallicities. The abundance of Co in this star is unusually low for its metallicity. What stands out is the large variance between the odd-*Z* and even-*Z* elemental abundances, the so-called odd–even effect, such as Na/Mg and Co/Ni. The absence of absorption lines of neutron-capture elements such as Sr and Ba in J1010+2358 is also notable. The upper limits of the abundances of Sr and Ba are lower than expected for a VMP star. This implies that there is no evidence for enrichment of rapid or slow neutron-capture process elements^13^.

**Table 1:** Abundance results obtained for J1010+2358

Element	*N*	log *ε*	[X/Fe]	*σ*_random_ (dex)	*σ*_total_ (dex)
Na I	1	<+1.80	<−2.02	…	…
Mg I	3	+4.52	−0.66	0.10	0.12
Si I	2	+5.24	+0.15	0.08	0.10
Ca I	9	+3.79	−0.13	0.05	0.08
Sc II	1	<−0.56	<−1.28	…	…
Ti I	3	+2.03	−0.50	0.12	0.14
Ti II	4	+1.88	−0.65	0.11	0.14
Cr I	6	+2.79	−0.43	0.06	0.13
Mn I	4	+2.15	−0.86	0.05	0.13
Fe I	60	+5.08	0.00	0.06	0.12
Fe II	7	+5.03	−0.05	0.07	0.12
Co I	1	+1.85	−0.72	0.06	0.12
Ni I	3	+3.63	−0.17	0.06	0.09
Zn I	1	<+1.89	<−0.25	…	…
Sr II	1	<−1.80	<−2.25	…	…
Ba II	1	<−1.61	<−1.37	…	…

The elemental abundances are expressed with respect to hydrogen (log *ε*(X) = log(*N*_X_/*N*_H_) + 12). The abundance of Fe I is adopted for calculating [X/Fe] ratios ([A/B] = log(*N*_A_/*N*_B_)_star_ − log(*N*_A_/*N*_B_)_⊙_, in which *N*_A_ and *N*_B_ are the number density of elements of A and B, respectively, the subscript ⊙ represents the solar value^29^). *N* is the number of lines analysed for the given species. The adopted atmospheric parameters are effective temperature *T*_eff_ = 5,860 ± 120 K, surface gravity in cgs units log *g* = 3.6 ± 0.2 and microturbulent velocity *v*_t_ = 1.5 ± 0.25 km s^−1^. *σ*_random_ is the standard deviation of abundances derived from individual lines. The errors induced by the uncertainties of atmospheric parameters are added in quadrature to *σ*_random_ for deriving the total error.

**Fig. 2:**
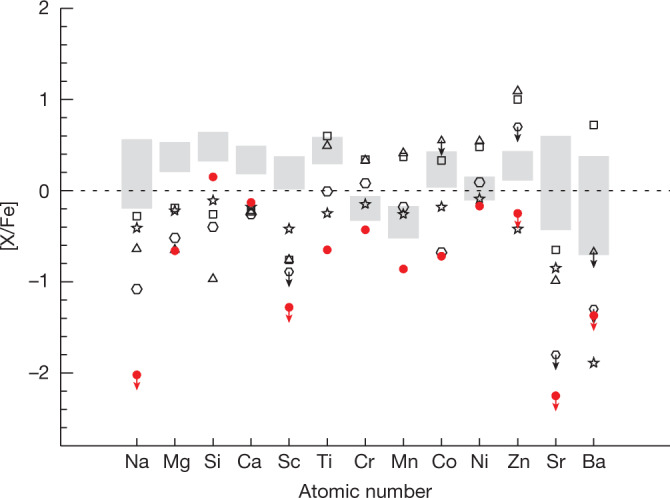
Abundance pattern of J1010+2358. The red circles denote J1010+2358. The open symbols indicate four previously known metal-poor stars with sub-solar [Mg/Fe] ratios. The abundances of these α-poor metal-poor stars (−2.46 ≤ [Fe/H] ≤ −1.91) have been well studied on the basis of high-resolution spectroscopic analysis^15,16^. The shaded regions indicate abundances of other metal-poor stars from the literature^10,11^. The arrows represent the upper limits.

The metal-poor stars in the Galactic halo typically possess enhanced α-element abundances ([Mg/Fe] > +0.3) owing to the chemical enrichment with core-collapse supernovae (CCSNe, enhancement of α elements) and the absence of Type Ia supernova (SN Ia) contributions^14^ (enhancement of iron). The low abundances of α elements with respect to iron in J1010+2358 show an excessive enrichment of iron. A few metal-poor stars are known to have low α element to iron ratios (α-poor stars)^15,16^ that are similar to J1010+2358, but none of these stars exhibits such low abundances of iron peak [X/Fe] (for example, Cr, Mn, Co, Ni and Zn) as J1010+2358 (Fig. 2). The model at present ^14^,^16^,^17^ is that the abundance patterns of previously known α-poor stars are the result of large iron yields from SN Ia. Combined with the enrichment of α elements (for example, Mg, Si and Ca) by CCSNe^18^, the contribution of SN Ia leads to the increase of iron-peak elements only and, thereby, to the decrease of [α/Fe] ratio^19^. As shown in Fig. 2, the previously known α-poor stars present normal or higher abundances of [Cr/Fe] and [Mn/Fe], along with low α element to iron ratios. By contrast, the abundances of [Cr/Fe] and [Mn/Fe] in J1010+2358 are much lower than those of other stars, ruling out any contribution from SN Ia. In general, the peculiar abundance pattern of J1010+2358 is markedly different from any known stars. Its abundance pattern is not likely to be produced by nucleosynthetic yields of several progenitors, as contributions from normal nucleosynthesis (for example, core-collapse supernova (CCSN) or SN Ia) would obscure such a peculiar feature of chemical abundances. The entire abundance pattern could be produced by nucleosynthesis from a very massive first-generation star, which contributes excess iron into the interstellar medium by means of a PISN^5^.

**Fig. 3:**
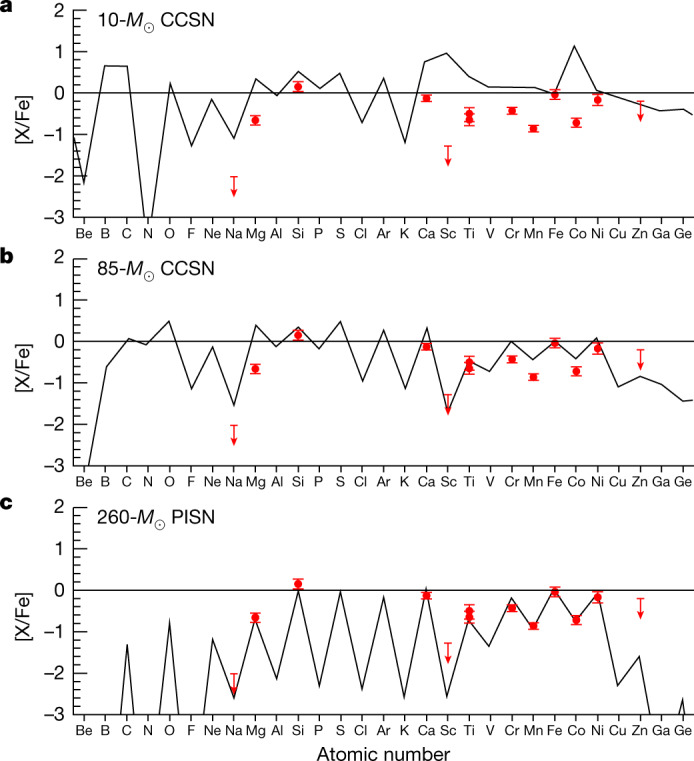
Comparison of observed abundances and models. The chemical abundances of J1010+2358 compared with the predictions from three theoretical supernova models^5,18^: a 10-*M*_⊙_ CCSN (**a**); a 85-*M*_⊙_ CCSN (**b**); a 260-*M*_⊙_ PISN with a 130-*M*_⊙_ He core (**c**). The error bars are 1*σ* uncertainties of the observed abundances.

We compare the observed abundance pattern of J1010+2358 with theoretical predictions on nucleosynthesis yields of CCSNe and PISNe (Fig. 3). The evolution of massive first stars with initial masses of about 10–140 *M*_⊙_ is considered to lead to iron-core collapse at the end to explode as CCSNe. The non-rotating stars with helium core masses of about 65–130 *M*_⊙_ (corresponding to initial masses of zero-age main-sequence massive stars of 140–260 *M*_⊙_) are expected to lead to the production of electron–positron pairs (e^+^/e^−^) before oxygen ignition, causing rapid contraction and the ignition of explosive oxygen burning. This process finally leads to an energetic thermonuclear runaway that is referred as a PISN, ejecting a large amount of heavy elements and leaving no remnant behind. The PISNe require their progenitors to have a helium core with a mass greater than 65 *M*_⊙_, which can only be fulfilled at extremely low metallicity because a very massive star triggers too strong stellar wind mass loss at high metallicity to form a massive helium core.

As shown in Fig. 3, the observed ratio of [Mg/Fe] and the upper limit of [Na/Fe] in J1010+2358 are much smaller than those from theoretical predictions of CCSNe, which exclude the possibility that the observed abundance pattern of J1010+2358 results from nucleosynthesis of a CCSN. Furthermore, the low [Cr/Fe], [Mn/Fe] and [Co/Fe] ratios are inconsistent with the expectation of CCSNe. The PISN models^5^ for zero-metallicity non-rotating progenitors with initial masses of 140–260 *M*_⊙_ that were computed by using the one-dimensional implicit hydrodynamics code KEPLER^20,21^ are also compared with the abundance pattern of J1010+2358 for exploring the enrichment source. The nucleosynthetic yields predicted by the PISN model with an initial mass of 260 *M*_⊙_ can closely reproduce the observed abundance pattern of J1010+2358 (Fig. 3). The odd–even effect in PISNe is substantially larger than that in CCSNe (ref. ^22^), which agrees well with the fact that J1010+2358 exhibits a stronger odd–even effect compared with other metal-poor stars in the Milky Way. The absence of neutron-capture elements in J1010+2358 is also in good agreement with the expectation of low-metallicity PISNe. Given the lack of heavy seed nuclei and appreciable neutron sources in helium burning, no *s*-process is expected in low-metallicity PISNe. Also, the production of *r*-process elements requires a very neutron-rich condition. The neutron deficiency is predicted in PISNe, which leads to the lack of *r*-process in PISNe. The low abundance ratios of [Na/Fe], [Mg/Fe], [Mn/Fe] and [Co/Fe] seen in J1010+2358 strongly suggest a PISN contribution. The iron-peak elements in PISNe are mostly produced by the incomplete Si burning and the incomplete-Si-burning regions in PISNe are much smaller than those in CCSNe, which leads to a low production of Mn and Co in PISNe. The production of Na requires excess neutrons and it is very sensitive to the initial metallicity in PISNe. The neutronization during the final evolutionary stages in PISNe is much less notable than in CCSNe, leading to a remarkable deficiency of odd-charged nuclei compared with even-charged nuclei in the nucleosynthesis of PISNe. The yield of α elements such as Mg is expected to be inefficient for massive, low-metallicity PISN progenitor models.

The discovery of J1010+2358 has provided a clear chemical signature for the existence of PISNe from very massive first stars. Its metallicity ([Fe/H] = −2.42) shows that the second-generation stars formed in the material enriched by supernovae from the first massive metal-free stars do not have to be extremely metal poor ([Fe/H] < −3)^23–25^. The extremely metal-poor stars are formed in the pristine gas polluted by very few CCSNe from Population III stars^7,26^ with masses less than 100 *M*_⊙_. Given that such Population III stars (<100 *M*_⊙_) live longer than the progenitors (140–260 *M*_⊙_) of PISNe, the second-generation stars with relatively high metallicities ([Fe/H] > −3), such as J1010+2358, should be formed in PISN-dominated cloud before the birth of the most metal-poor stars with CCSN imprints. Notably, a very low [Mg/Fe] as found for J1010+2358 has been observed in a broad line region in a very-high-redshift quasar^27^ with a high [Fe/H], for which a large amount of iron contributed by PISNe is suggested. The peculiar abundances of J1010+2358 provide key features for identifying PISN signatures. Detailed studies of VMP stars included in the large stellar abundance databases^28^ will facilitate the discovery of more PISN-dominated stars and provide an essential clue to constraining the initial mass function in the early universe.

## Methods

### Observational data and stellar parameters

The high-resolution (*R* = 36,000) spectroscopic observation of LAMOST J1010+2358 was obtained by using Subaru/High Dispersion Spectrograph (HDS)^30^ on 3 June 2015. The high-resolution spectra cover the wavelength range of 4,000–6,800 Å with a gap of 5,330–5,430 Å. The resolving power of *R* ≈ 36,000 is obtained by using a 1.0-arcsec slit and 2 × 2 CCD pixel binning. The signal-to-noise ratios at 4,300 and 5,000 Å are 50 and 70, respectively. Data reduction, including bias correction, flat fielding, scattered light subtraction and wavelength calibration, was carried out with the IRAF echelle package.

The radial velocity of J1010+2358 was measured from Fe I lines that are used for abundance analysis. The heliocentric radial velocity derived from the high-resolution spectra, −101.8 ± 0.7 km s^−1^, are in good agreement with that from the LAMOST spectra. The EWs of isolated absorption lines were measured by fitting Gaussian profiles with the IRAF task splot by using a line list compiled from the literature^31,32^. The kinematic analysis indicates that this star is a Galactic halo star on a retrograde orbit.

Stellar parameters, including effective temperature (*T*_eff_), surface gravity (log *g*) and microturbulent velocity (*v*_t_), are determined spectroscopically from isolated absorption lines of Fe based on the LTE model atmospheres^12^. The abundances of individual Fe I and Fe II lines are derived by using the MOOG program^33^. *T*_eff_ is determined by forcing the abundances derived from individual Fe I lines to be independent of their excitation potential. We also estimate *T*_eff_ from the (V–K)_0_ colours^34^ (*T*_eff_ = 5,810 K), which is in close agreement with that from spectroscopic analysis. Surface gravity is derived from ionization equilibrium between Fe I and Fe II. Microturbulent velocity is estimated from individual Fe I lines by requiring the derived abundances to be independent of their EWs.

### Abundance determination

**Extended Data Fig. 1:**
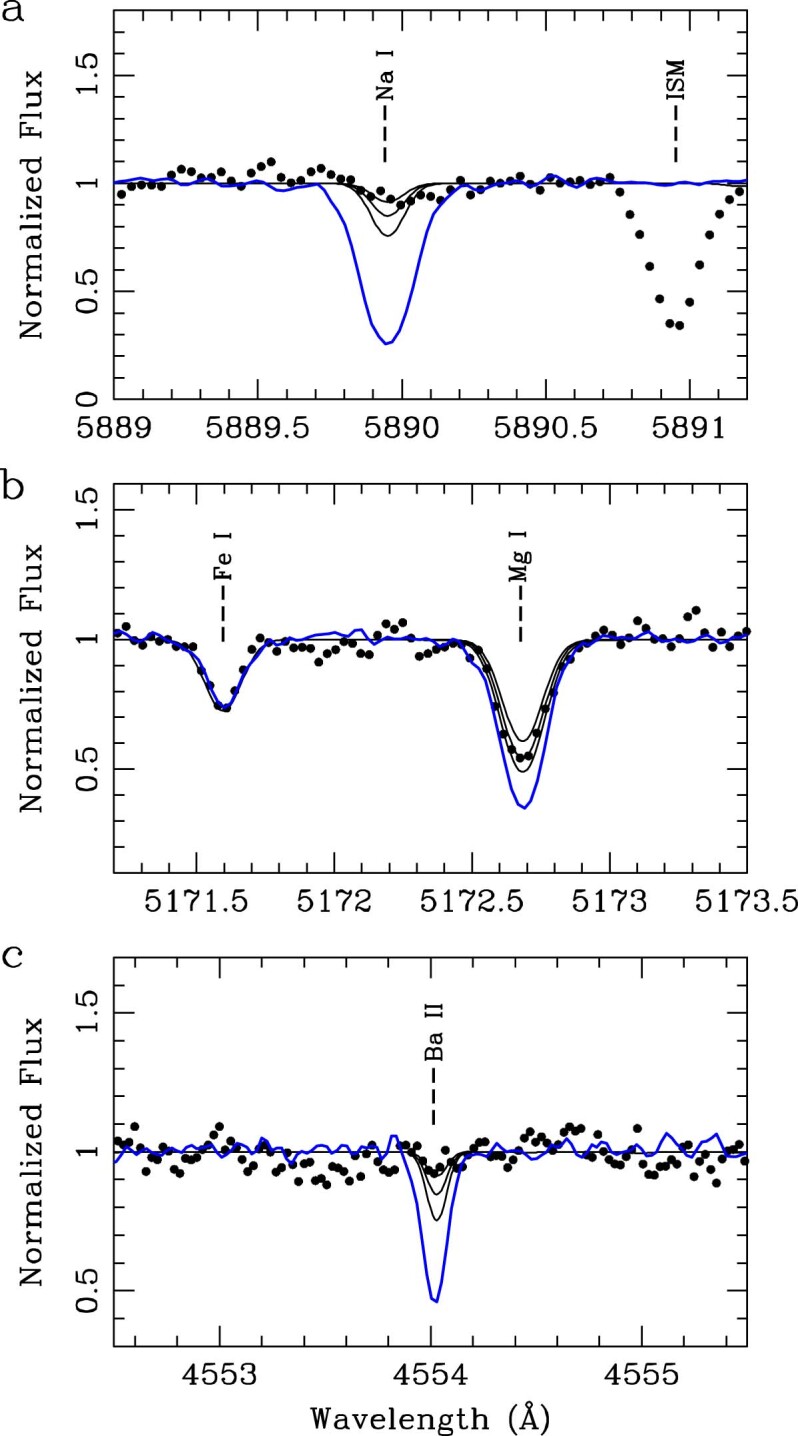
Observed spectrum of J1010+2358. The observed spectrum of J1010+2358 (filled circles: *T*_eff_ = 5,860 K; log *g* = 3.6; [Fe/H] = −2.42) are shown with a comparison star J0626+6032 (blue lines: *T*_eff_ = 5,863 K; log *g* = 3.73; [Fe/H] = −2.39). The chemical abundances ([Na/Fe] = +0.89 ± 0.08; [Mg/Fe] = +0.21 ± 0.06; [Ba/Fe] = +0.23 ± 0.06) of J0626+6032 are normal as a VMP star. The black lines are synthetic spectra with [Na/Fe] = −2.02 ± 0.3 (**a**), [Mg/Fe] = −0.66 ± 0.3 (**b**) and [Ba/Fe] = −1.37 ± 0.3 (**c**).

The abundances of most elements lighter than Zn are determined from EWs based on the adopted stellar parameters. The upper limits of Na, Sc, Zn, Sr and Ba abundances are estimated by spectrum synthesis. Furthermore, the abundances of elements other than Fe determined from EWs analysis are also confirmed by spectrum synthesis. For lines of Sc II, Mn I and Co I, the effect of hyperfine splitting is included in the abundance determination by using the data in Kurucz’s database. Both of the Na I lines at 5,889 Å and 5,895 Å are too weak for EW measurements. The upper limit of Na abundance is determined from synthesis of the Na I 5,889 Å line. We notice that the Na abundance ([Na/Fe] < −2.02) of J1010+2358 is extremely low compared with other metal-poor stars. A portion of the spectrum of a comparison star LAMOST J0626+6032 (*T*_eff_ = 5,863 K, log *g* = 3.73, [Fe/H] = −2.39, [Na/Fe] = +0.89) is shown in Extended Data Fig. 1 for comparison purposes. The spectrum of the comparison star was obtained by Subaru/HDS with the same setup. The α elements with detectable lines include Mg, Si, Ca and Ti. The Mg lines at 4,702, 5,172 and 5,183 Å were used to determine Mg abundance. The [X/Fe] ratios for Mg, Ca and Ti are sub-solar, whereas [Si/Fe] is slightly enhanced. No absorption line of neutron-capture elements is detected in the spectrum. The upper limits of Sr and Ba are estimated from Sr II 4,077 Å and Ba II 4,554 Å, respectively. The portion of the spectrum of J1010+2358 around Ba II 4,554 Å is shown in Extended Data Fig. 1 for comparison. The carbon feature could not be detected from the molecular band of CH at 4,315 Å. Non-LTE corrections are estimated for lines of Na, Mg, Si, Ca, Cr, Mn and Fe (refs. ^35–38^). The non-LTE corrections for Na I, Mg I, Si I, Mn I and Fe I are less than +0.1 dex, whereas the corrections for Ca I and Cr I are +0.16 dex and +0.21 dex, respectively. The PISN model is still the best-fitting model when the non-LTE corrections are included in the comparison between the observed abundance pattern and SN yield models.

### Exclusion of CCSN models

The search algorithm STARFIT^18^ compares the observed abundances of J1010+2358 against a large number of SN yield models in the literature^39–44^ and determines that the best-fitting model is a 260-*M*_⊙_ PISN with a 130-*M*_⊙_ He core. One of the most important features of J1010+2358 is that its [Na/Fe] and [Mg/Fe] ratios are much lower than those of other metal-poor stars in the Milky Way with similar metallicities. It is impossible to reproduce such low [Na/Fe] and [Mg/Fe] abundance pattern by assuming the contributions from CCSNe because regular CCSNe cannot ever produce very low [α/Fe] ratio^45^. CCSNe with a higher explosion energy seem to be able to reduce the ratios of [Na/Fe] and [Mg/Fe] (even though it is still hard to achieve such low yields observed in J1010+2358), but they produce too high [Si/Fe] and [Co/Fe] to be consistent with the observations of J1010+2358 (refs. ^15,45^). Therefore, the possibility of CCSNe as the enrichment source of J1010+2358 can be excluded.

### Exclusion of SN Ia contribution

**Extended Data Fig. 2:**
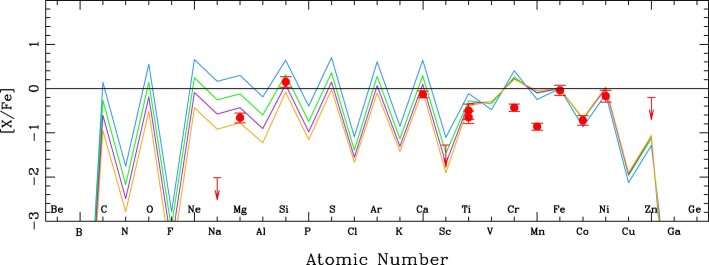
Comparison of observed abundance pattern and SN yield models. The chemical abundances of J1010+2358 (red circles) compared with models of combinations of SN Ia (ref. ^46^) and CCSN (refs. ^15,51^) with mass of 25 *M*_⊙_: 10% contribution from SN Ia (blue line); 30% contribution from SN Ia (green line); 50% contribution from SN Ia (purple line); 70% contribution from SN Ia (orange line). The error bars are 1*σ* uncertainties of the observed abundances.

The previously known α-poor stars have been generally explained by the nucleosynthetic yields of SN Ia along with a contribution from a CCSN. The combination of a SN Ia and a normal CCSN is expected to produce a low [α/Fe] ratio because of the iron enhancement resulting from SN Ia, which is required for explaining the low [α/Fe] ratio observed in J1010+2358. However, the low [Na/Fe], [Cr/Fe] and [Mn/Fe] ratios observed in J1010+2358 are in conflict with the expectation of the yields of SN Ia models^46,47^ (Extended Data Fig. 2). It is noted that a combination of hypernovae^48^ and sub-Chandrasekhar-mass SNe Ia (refs. ^49,50^) may produce a lower [Mn/Fe]. But the theoretical predictions of [Si/Fe], [Ti/Fe] and [Co/Fe] from such a combination are substantially different from the observed abundances of J1010+2358. Moreover, the long interval between the two progenitors would result in enrichment from normal CCSNe, which is not consistent with the observed abundance pattern of this star.

## Online content

Any methods, additional references, Nature Portfolio reporting summaries, source data, extended data, supplementary information, acknowledgements, peer review information; details of author contributions and competing interests; and statements of data and code availability are available at 10.1038/s41586-023-06028-1.

## Data Availability

The data used in this analysis are available on the archive of the Japanese Virtual Observatory (http://jvo.nao.ac.jp/portal/top-page.do).

## Code availability

The MOOG code for stellar line analysis and spectrum synthesis is available at https://www.as.utexas.edu/~chris/moog.html. The STARFIT tool for finding the best-fitting SN yield model is available at https://starfit.org.
